# CAM use among overweight and obese persons with radiographic knee osteoarthritis

**DOI:** 10.1186/1472-6882-13-241

**Published:** 2013-09-28

**Authors:** Kate L Lapane, Shibing Yang, Rachel Jawahar, Timothy McAlindon, Charles B Eaton

**Affiliations:** 1 Department of Quantitative Health Sciences, University of Massachusetts Medical School, Worcester, MA, USA; 2 Department of Epidemiology and Community Health, Virginia Commonwealth University, Richmond, VA, USA; 3 Department of Rheumatology Tufts Medical School, Boston, MA, USA; 4 Center for Primary Care and Prevention, Memorial Hospital of Rhode Island, Pawtucket, RI, USA; 5 Department of Family Medicine, Warren Alpert Medical School, Brown University, Providence, RI, USA

**Keywords:** Knee osteoarthritis, Obesity, Pain, Complementary and alternative medicine

## Abstract

**Background:**

Obesity is associated with knee pain and is an independent predictor of incident knee osteoarthritis (OA); increased pain with movement often leads patients to adopt sedentary lifestyles to avoid pain. Detailed descriptions of pain management strategies by body mass index (BMI) level among OA patients are lacking. The objectives were to describe complementary and alternative medicine (CAM) and conventional medication use by BMI level and identify correlates of CAM use by BMI level.

**Methods:**

Using Osteoarthritis Initiative baseline data, 2,675 patients with radiographic tibiofemoral OA in at least one knee were identified. Use of CAM therapies and conventional medications was determined by interviewers. Potential correlates included SF-12, CES-D, Western Ontario and McMaster Universities Osteoarthritis Index, and Knee injury and Osteoarthritis Outcome Score quality of life. Multinomial logistic regression models adjusting for sociodemographic and clinical factors provided estimates of the association between BMI levels and treatment use; binary logistic regression identified correlates of CAM use.

**Results:**

BMI was inversely associated with CAM use (45% users had BMI ≥35 kg/m^2^; 54% had BMI <25 kg/m^2^), but positively associated with conventional medication use (54% users had BMI ≥35 kg/m^2^; 35.1% had BMI <25 kg/m^2^). Those with BMI ≥30 kg/m^2^ were less likely to use CAM alone or in combination with conventional medications when compared to patients with BMI <25 kg/m^2^.

**Conclusions:**

CAM use is common among people with knee OA but is inversely associated with BMI. Understanding ways to further symptom management in OA among overweight and obese patients is warranted.

## Background

Obesity is an independent predictor of incident knee osteoarthritis (OA) [1]. Both weight gain and malalignment are also associated with increased pain and functional loss [2]. OA patients with body mass index (BMI) ≥ 35 kg/m^2^ often experience increased pain due to significant increases in joint stress and load forces on the knees [3]. Obesity is a modifiable risk factor for the development and treatment of knee pain [4]. In two major trials [5,6], people randomized to intensive lifestyle interventions which focused on exercise and weight loss demonstrated improvements in pain and physical function relative to controls. Interventions including both exercise and weight loss were more successful than those using either approach alone [7]. Diffusing interventions is challenging because increased pain with movement often leads patients to adopt sedentary lifestyles to avoid pain, which leads to more weight gain, pain, and disability.

OA is a chronic disease with no cure so patients often treat pain with conventional medications or therapies [8] and complementary and alternative medicines (CAM) [9,10]. Glucosamine [11] and acupuncture [12] do relieve symptoms among OA patients. Obese adults are less likely to use CAM overall [13], but detailed CAM practices among people with higher BMI are unknown.

The Osteoarthritis Initiative (OAI) provides the opportunity to address this gap in the literature. The OAI is a multi-center, prospective observational study which examines the natural history of and identifies risk factors for incidence and progression of knee OA [14]. The OAI is a unique data source because it provides a population with radiographic confirmation of OA and detailed assessments of knee-specific pain, quality of life, and functioning indicators using standardized instruments. The study purpose was twofold: 1) to describe differences in treatment approaches to manage symptoms of knee OA by level of BMI; and 2) to evaluate the extent to which sociodemographic and clinical correlates of CAM use differed by BMI.

## Methods

The University of Massachusetts Medical School Institutional Review Board reviewed and approved the protocol for this study. Because publicly-available data were used for this study, the Institutional Review Board waived the need for documentation of informed consent from participants.

We used publicly available data from the OAI (http://www.oai.ucsf.edu/) (#AllClinical00, V0.2.2). The OAI began recruiting in 2004 and engaged 4,796 participants aged 45 to 79 years. At baseline, each participant underwent 3.0 Tesla MRI examinations of the knee and provided blood samples, and each clinical site had readers (trained through didactic and interactive web-based methods) assess fixed flexion knee x-rays for osteophytes and joint space narrowing. Eligibility was restricted to those without severe joint space narrowing in both knees. The participants were followed annually for the development or progression of knee OA. We included 2,679 individuals with radiographic tibiofemoral knee OA (e.g. OARSI atlas osteophyte grade I–III) [15] in at least one knee at baseline and excluded 4 participants with missing height and/or weight measured using standardized methods (n = 2,675). Participants were classified into four categories: BMI less than 25 kg/m^2^, BMI between 25 and less than 30 kg/m^2^ (overweight), BMI from 30 to less than 35 kg/m^2^ (obese), and BMI of at least 35 kg/m^2^ (severe obesity) [16].

### Classification of use of CAM and conventional medications

Complementary and alternative therapies were defined as [9]: 1) alternative medical systems (e.g. homeopathy, acupuncture); 2) mind-body interventions (e.g. pilates, spiritual activities, relaxation therapy); 3) manipulation and body-based methods (e.g. massage and chiropractic); 4) energy therapies (e.g. copper bracelets); 5) topical biologically based therapies including rubs (e.g. tiger balm); 6) biologically based diet; or 7) biologically based supplements (e.g. glucosamine, chondroitin). CAM use for the past year was determined by a series of questions including, “During the past 6 months, did you use the following health supplements for joint pain or arthritis?” Conventional medication use was captured in baseline surveys as self-reported use. A four-level outcome variable was created: CAM use only, conventional medication use only, both, and no use.

### Potential correlates

Treatment of OA is influenced by sociodemographic indicators, overall measures of mental and physical well-being, and clinical indices of OA. We anticipated CAM use to be different by age group [17], gender [18], race/ethnicity [19,20], education [21], annual household income, employment status, and health insurance status. Physical and mental health status were assessed by the 12-item Medical Outcomes Study Short Form (SF-12) [22] (range from 0 to 100, with higher scores indicating better health status). Depression status was measured with the CES-D Scale (≥16) [23].

A pain score of 20 in the Western Ontario and McMaster Universities (WOMAC) Osteoarthritis Index (Version LK 3.1) [24,25] indicated the worst pain (range 0 to 20). Knee-related quality of life was measured by the Knee injury and Osteoarthritis Outcome Score (KOOS) by calculating a summary score ranging from 0 to 100 (range: 0 (extreme symptoms) to 100 (no symptoms)) [26]. The knee with worse measures was used in the analysis. Walking ability and endurance were measured by a 20-meter walk, averaged over two trials [27]. The chair stand test directly assessed leg strength and knee function and duration of time (seconds) needed to stand up and sit down five times as quickly as possible [28].

Participants were classified by x-ray joint space narrowing as determined by OARSI atlas grade on a fixed flexion radiograph of the knee with the worst measure. Multiple-joint OA symptoms were measured with self-reported information at baseline: low back pain in previous 30 days, OA in hand, hip symptoms, and hip replacement. Information on previous history of knee injury or surgery was also collected.

### Analytic approach

A multivariable multinomial logistic regression model was developed to estimate the association between BMI level and CAM/conventional treatment use after adjusting for sociodemographic and clinical characteristics. We used a multinomial logistic regression model because the outcome variable of interest was four levels: 1) use of CAM only; 2) use of conventional medications only; 3) use of both; and 4) use of neither. The models produced odds ratios (ORs) and corresponding 95% confidence intervals (CIs). Multicollinearity among the variables of interest was assessed and ruled out by evaluating a correlation matrix before the modeling process and then by carefully evaluating the standard errors as new variables were introduced into the model. Odds ratios for the KOOS- QOL scale and chair stand test were calculated as one standard deviation change in each variable. To determine correlates of CAM use stratified by BMI levels, we created separate logistic regression models for each BMI level. The outcome variable in these models was use of CAM (yes/no).

## Results

Table 1 shows sociodemographic measures by BMI level. Most participants with BMI ≥ 35 kg/m^2^ were women (69.0%) and were younger than participants with BMI < 25 kg/m^2^ (27.5% over 65 years of age vs. 49.9%). Seventy-four percent of participants with BMI < 25 kg/m^2^ were married whereas 53.4% of those with BMI ≥ 35 kg/m^2^ were. Sixty-six percent of participants with BMI < 25 kg/m^2^ were at least a college graduate whereas 41% of those with BMI ≥ 35 kg/m^2^ had.

**Table 1:** Sociodemographic and descriptive characteristics of participants with radiographic-confirmed knee OA by BMI level (N = 2,675)

	**BMI ≥ 35 kg/m**^**2**^	**BMI between 30 and <35 kg/m**^**2**^	**BMI between 25 and <30 kg/m**^**2**^	**BMI < 25 kg/m**^**2**^
	**(n = 364)**	**(n = 804)**	**(n = 1,042)**	**(n = 465)**
	*Percentage*			
Age (years): ≥ 65	27.5	39.6	48.9	49.9
Women	69.0	57.8	50.3	69.0
Race/ethnicity: White	58.8	72.7	82.7	89.3
African American	38.7	24.3	13.3	6.5
Latino	1.1	0.8	1.8	1.7
Other	1.4	2.2	2.1	2.6
Education:≥College graduate	40.8	50.4	59.9	66.2
Some college	36.9	28.2	22.7	21.3
≤ High school	22.4	21.3	17.5	12.6
Income ($):>100,000	15.6	20.1	24.3	22.4
50 k–100 k	29.9	35.8	36.2	40.0
25 k–50 k	31.4	27.9	26.7	24.0
≤ 25,000	23.1	16.2	12.8	13.7
Married/partnered	53.4	62.9	68.8	73.9
Working (for pay)	65.1	61.1	57.0	52.3
Health insurance	91.6	96.1	98.3	98.5
Insurance covers prescriptions	83.3	87.5	87.9	86.7
CES-D > 16 (Depressed)	15.9	10.1	7.2	7.5
	*Mean (standard deviation)*			
Weight at age 25 (kg)	71.7 (14.9)	70.8 (14.7)	67.7 (13.1)	60.7 (10.1)
SF-12* Mental summary	52.3 (9.5)	53.4 (8.6)	54.7 (7.5)	53.9 (7.9)
SF-12* Physical summary	42.4 (10.6)	46.8 (9.3)	48.6 (9.0)	50.9 (8.0)

*SF-12 (range: 0 to 100 with higher scores indicating better health).

Table 2 shows clinical measures by BMI level. The mean KOOS QOL score for participants with BMI < 25 kg/m^2^ was 69.5 (standard deviation: 21.0) and it was 53.5 in participants with BMI ≥ 35 kg/m^2^ (standard deviation: 25.1). The mean WOMAC pain scores was 6.1 in participants with BMI ≥ 35 kg/m^2^ (standard deviation: 4.7) and 2.8 in participants with BMI < 25 kg/m^2^ (standard deviation: 3.3). Severe joint space narrowing was 20.9% in participants with BMI ≥ 35 kg/m^2^ and 16.8% in participants with BMI < 25 kg/m^2^.

**Table 2:** Clinical characteristics of participants with radiographic-confirmed knee OA by BMI level (N = 2,675)

	**BMI ≥ 35 kg/m**^**2**^	**BMI between 30 and <35 kg/m**^**2**^	**BMI between 25 and <30 kg/m**^**2**^	**BMI < 25 kg/m**^**2**^
	**(n = 364)**	**(n = 804)**	**(n = 1,042)**	**(n = 465)**
**Symptoms**	*Mean (standard deviation)*			
WOMAC*–Pain	6.1 (4.7)	4.4 (4.0)	3.7 (3.6)	2.8 (3.3)
KOOS*–QOL	53.5 (25.1)	60.3 (22.5)	64.2 (22.5)	69.5 (21.0)
**Function and performance**				
Isometric strength/chair stands (seconds)	13.1 (4.4)	12.1 (4.0)	11.6 (3.8)	10.6 (3.0)
20-meter walk (seconds)	17.5 (4.0)	16.3 (3.1)	15.5 (2.8)	15.1 (2.6)
**Joint space narrowing: x-ray evidence of knee severity**	*Percentage*			
OARSI grade 0 (normal)	26.9	27.5	31.0	35.1
OARSI grade 1–2 (narrowed)	52.2	51.5	46.6	48.2
OARSI grade 3 (severe)	20.9	21.0	22.4	16.8
**Multi-joint osteoarthritis**				
Bilateral knee OA	68.4	66.0	60.9	53.3
Any back pain (30 days)	66.5	56.7	56.3	59.6
Hand osteoarthritis	15.8	17.6	18.1	22.5
Hip symptoms (12 months)	30.9	24.4	23.9	21.2
Total hip replacement	0.8	2.5	1.8	1.9
**History**				
History of knee injury	52.8	46.8	47.2	46.0
History of knee surgery	27.6	29.0	31.9	26.9

*WOMAC score ranges from 0 to 20, with 20 indicating worst pain. KOOS score ranges from 0 (extreme symptoms) to 100 (no symptoms).

Table 3 shows specific CAM therapies stratified by BMI level. Fifty percent of participants with BMI < 25 kg/m^2^ and 39.6% of participants with BMI ≥ 35 kg/m^2^ used one or two CAM therapies, either alone or with conventional medication. Although more participants with BMI < 25 kg/m^2^ used CAM overall relative to overweight and obese participants, differences by specific CAM type exist. Energy therapies were more common amongst overweight and obese participants (5.8% ≥ 35 kg/m^2^ versus 3.4% BMI < 25 kg/m^2^) as were topical therapies (18.1% ≥ 35 kg/m^2^ versus 9.7% BMI < 25 kg/m^2^). Table 4, which shows specific conventional therapies by BMI level, indicates the reverse is true for conventional medications; 8.4% of participants with BMI < 25 kg/m^2^ and 19% of participants with BMI ≥ 35 kg/m^2^ reported at least two conventional medications. Doxycycline use was very low overall.

**Table 3:** CAM use among participants with radiographic-confirmed knee OA by BMI level (N = 2,675)

**Category**^**a**^	**BMI ≥ 35 kg/m**^**2**^	**BMI between 30 and <35 kg/m**^**2**^	**BMI between 25 and <30 kg/m**^**2**^	**BMI < 25 kg/m**^**2**^
	**(n = 364)**	**(n = 804)**	**(n = 1,042)**	**(n = 465)**
	*Percentage*			
**Alternative medical systems**	1.1	0.8	1.1	2.2
Acupuncture	0.3	0.5	0.5	1.5
Acupressure	0.3	0	0.3	0.4
Chelation therapy	0	0	0	0
Folk medicine	0	0	0	0
Homeopathy	0.3	0	0.4	0.2
Ayurveda/biofeedback/energy healing/ hypnosis/naturopathy	0.8	0.3	0.3	0.4
**Mind-body interventions**	11.8	10.8	8.4	14.6
Yoga/Tai Chi/Chi Gong/Pilates	3.9	4.5	5.3	10.3
Relaxation therapy, meditation, breathing	3.6	4.6	2.5	3.9
Spiritual activities	7.1	4.4	2.7	3.2
**Manipulation and body-based methods**	5.5	5.7	4.9	5.0
Chiropractic	4.7	4.6	4.1	3.9
Massage	2.5	1.7	1.6	1.7
**Energy therapies (Copper bracelets or magnets)**	5.8	3.1	3.2	3.4
**Biologically based therapies: topical agent**	18.1	15.1	11.9	9.7
Rubs, lotions, liniments, creams or oils(tiger balm/horse liniment)	17.6	14.8	11.7	9.7
Capsaicin	1.9	1.7	1.6	1.3
**Biologically based therapies: diet**	2.2	0.8	0.9	1.7
**Biologically based therapies: supplements**	23.4	29.2	32.9	41.3
Herbs	1.7	1.9	1.2	2.2
Vitamins/minerals (nearly every day)	5.2	6.0	6.3	7.5
Glucosamine (nearly every day)	19.8	24.5	28.8	36.6
Methylsulfonylmethane (MSM)	3.6	6.0	6.0	6.5
S-adenosylmethionine (SAMe)	0.0	0.3	0.7	0.9
Chondroitin (nearly every day)	19.0	22.1	26.5	32.5
**Distribution**^**b **^**of CAM use:**				
One	28.3	32.0	33.0	35.1
Two	11.3	9.8	9.1	14.4
Three or more	5.0	4.2	3.5	4.3

^a^As defined by the National Center for Complementary and Alternative Medicine;

^b^Number of CAM use was defined on basis of the broader categories.

**Table 4:** Conventional medication use among participants with radiographic-confirmed knee OA by BMI level (N = 2,675)

**Category**	**BMI ≥ 35 kg/m**^**2**^	**BMI between 30 and <35 kg/m**^**2**^	**BMI between 25 and <30 kg/ m**^**2**^	**BMI < 25 kg/m**^**2**^
	**(n = 364)**	**(n = 804)**	**(n = 1,042)**	**(n = 465)**
	*Percentage*			
Acetaminophen	18.1	11.4	9.7	8.8
Any NSAIDs	35.4	26.9	24.2	23.2
Over-the-counter NSAIDs	27.8	21.4	19.7	18.1
Prescription NSAIDs	12.4	7.8	6.2	6.5
COX-2 Inhibitors	9.9	8.5	8.7	6.9
Acetaminophen or NSAIDs	45.9	34.1	29.9	28.4
Doxycycline	0.3	0.4	0.5	0.0
Narcotics	5.8	3.5	1.4	2.2
Knee injections^a^	4.7	4.5	3.4	2.6
Hyaluronic acid	1.1	1.1	1.2	0.9
Steroids	4.4	3.7	2.0	1.3
Distribution of conventional medications:^b^				
One	34.6	29.5	27.1	26.7
Two	13.5	9.1	7.9	6.9
Three or more	5.5	3.0	2.1	1.5

^a^The sum of percentages of hyaluronic acid and steroid injections may not be equal to the total percentage of knee injections because participants may use both hyaluronic acid and steroids, or use injections other than hyaluronic acid and steroids.

^b^Number of conventional medication used was based on the first seven items in this table.

**Figure 1:**
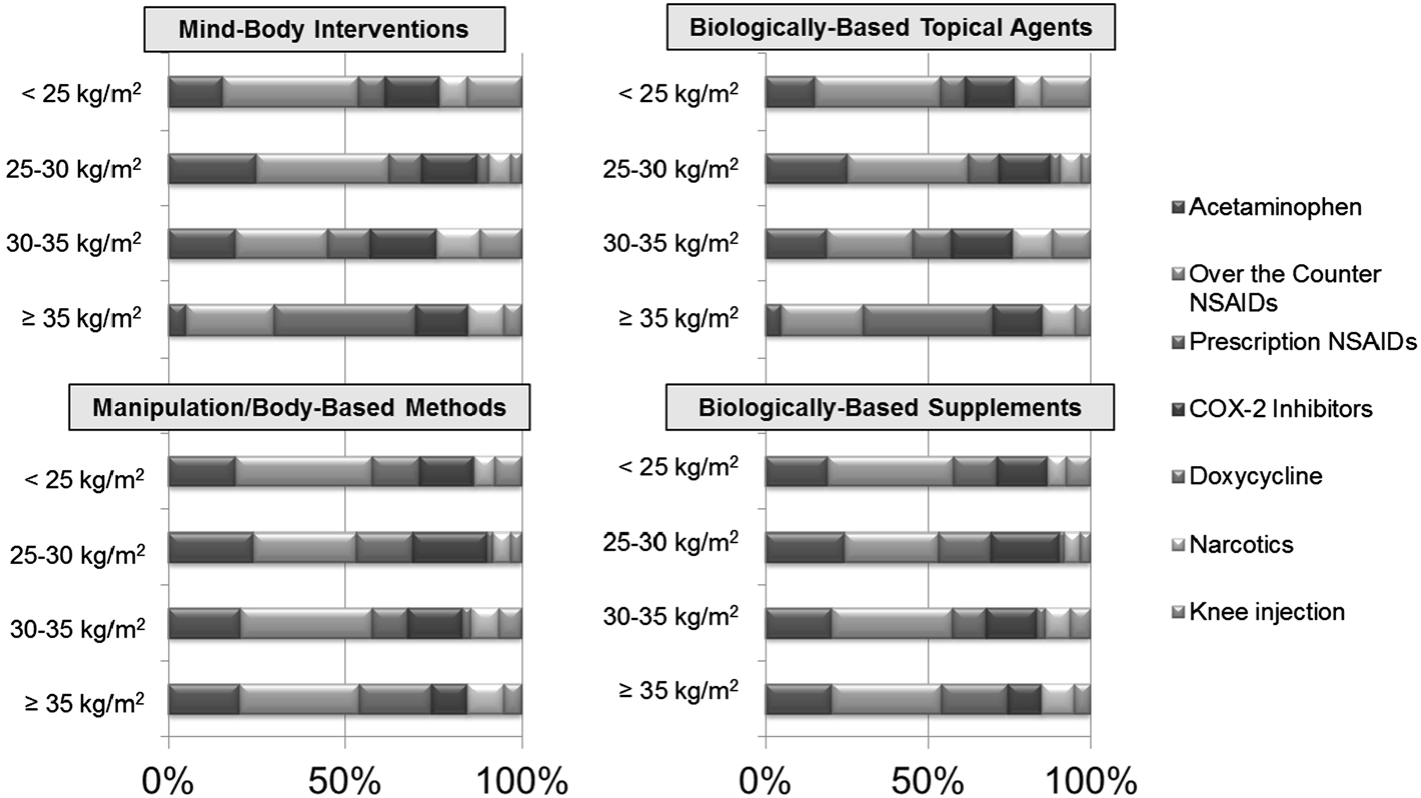
Distribution of concomitant conventional medication use among CAM users, stratified by BMI level.

Figure 1 shows CAM use and types of concurrent conventional medication use by BMI level. The most commonly used concurrent conventional therapies amongst all BMI groups was acetaminophen and/or non-steroidal anti-inflammatory agents (NSAIDs) which were over-the-counter or prescription.

Table 5 shows the association between CAM and conventional treatment use and BMI level. Both participants with BMI between 30 and 35 kg/m^2^ and participants with BMI between 25 and 30 kg/m^2^ reported using CAM with conventional medications less often than participants with BMI < 25 kg/m^2^ (adjusted odds ratio (AOR): 0.38, 95% CI: 0.20-0.73 for participants with BMI between 30 and 35 kg/m^2^; AOR: 0.35, 95% CI: 0.19-0.65 for participants with BMI between 25 and 30 kg/m^2^).

**Table 5:** Association between BMI level and treatment approaches among people with knee OA

**Treatment use**	**CAM only**	**Conventional medications only**	**Both**
	*Odds ratios*		
	*(95% Confidence intervals)*		
**BMI ≥ 35 kg/m**^**2 **^**versus referent group BMI <25 kg/m**^**2**^			
Crude	0.48	2.51	1.38
	(0.31–0.73)	(1.55–4.07)	(0.94–2.04)
Socio-demographic adjusted^†^	0.50	1.69	1.16
	(0.28–0.91)	(0.81–3.53)	(0.65–2.09)
Socio-demographic and clinical characteristic adjusted^††^	0.47	1.13	0.55
	(0.24–0.92)	(0.47–2.70)	(0.27–1.12)
**BMI between 30 and <35 kg/m**^**2 **^**versus referent group BMI <25 kg/m**^**2**^			
Crude	0.70	1.71	0.94
	(0.50–0.98)	(1.09–2.68)	(0.66–1.34)
Socio-demographic adjusted^†^	0.71	1.29	0.71
	(0.43–1.17)	(0.65–2.57)	(0.41–1.22)
Socio-demographic and clinical characteristic adjusted^††^	0.59	0.84	0.38
	(0.34–1.04)	(0.37–1.88)	(0.20–0.73)
**BMI between 25 and <30 kg/m**^**2 **^**versus referent group BMI <25 kg/m**^**2**^			
Crude	0.68	1.29	0.77
	(0.50–0.94)	(0.83–2.01)	(0.55–1.09)
Socio-demographic adjusted^†^	0.49	0.78	0.52
	(0.31–0.80)	(0.39–1.55)	(0.31–0.88)
Socio-demographic and clinical characteristic adjusted^††^	0.47	0.75	0.35
	(0.28–0.79)	(0.35–1.63)	(0.19–0.65)

^†^Adjusted for age, gender, race/ethnicity, marital status, education, employment status, income, health insurance, prescription drug insurance, and depression.

^††^Also adjusted for physical and mental health component scores, KOOS quality of life, WOMAC pain scale, weight at 25 years of age, hip replacement, history of knee surgery, complaints of pain in multiple joints, OARSI severity scale, isometric strength/chair stands, and 20-meter walk.

Table 6 shows correlates of any CAM use by BMI level. Participants with BMI ≥ 35 kg/m^2^ and over 65 years of age were more likely to use CAM than younger participants (AOR: 2.79, 95% CI: 1.53-5.09). Black persons with BMI between 25 and 30 kg/m^2^ were less likely to report CAM use compared to white persons (AOR: 0.36, 95% CI: 0.23-0.58). Among persons with BMI of 25 to 30 and 30 to 35 kg/m^2^, having a college degree or higher was associated with CAM use, relative to a high school education or less (AOR: 1.71 and 1.84, respectively).

**Table 6:** Correlates of CAM use among participants with radiographic-confirmed knee OA by BMI level

	**BMI ≥ 35 kg/m**^**2**^	**BMI between 30 and <35 kg/m**^**2**^	**BMI between 25 and <30 kg/m**^**2**^	**BMI < 25 kg/m**^**2**^
	**(n = 364)**	**(n = 804)**	**(n = 1,042)**	**(n = 465)**
	*Odds ratios (95% Confidence intervals)*^a^			
Age ≥ 65 years	2.79 (1.53-5.09)	1.29 (0.89-1.86)	1.01 (0.73-1.39)	1.22 (0.72-2.06)
Women	1.85 (1.08-3.15)	1.56 (1.11-2.17)	1.66 (1.25-2.21)	3.27 (2.03-5.27)
Race/ethnicity				
Black	0.87 (0.51-1.48)	0.84 (0.57-1.25)	0.36 (0.23-0.58)	1.06 (0.42-2.71)
Latino	1.04 (0.13-8.13)	1.73 (0.24-12.34)	0.98 (0.33-2.97)	1.53 (0.30-7.68)
Other	0.20 (0.02-2.14)	1.69 (0.57-5.00)	1.48 (0.54-4.07)	2.35 (0.63-8.81)
Non-hispanic white	1.0	1.0	1.0	1.0
Education				
≥College graduate	0.96 (0.49-1.87)	1.84 (1.20-2.82)	1.71 (1.14-2.56)	1.58 (0.80-3.13)
Some college	1.00 (0.52-1.91)	1.54 (0.97-2.43)	1.43 (0.91-2.24)	1.37 (0.63-2.96)
High school or less	1.0	1.0	1.0	1.0
Employment status	2.21 (1.26-3.88)	1.08 (0.75-1.55)	0.83 (0.61-1.13)	1.66 (1.01-2.73)
Depression	1.28 (0.66-2.50)	0.89 (0.52-1.52)	0.95 (0.55-1.64)	0.34 (0.14-0.81)
KOOS–QOL^b^	0.70 (0.53-0.91)	0.55 (0.45-0.66)	0.66 (0.57-0.78)	0.62 (0.48-0.80)
Multi-joint osteoarthritis	1.36 (0.79-2.33)	1.64 (1.17-2.30)	1.25 (0.94-1.68)	1.96 (1.24-3.11)
Isometric strength/chair stands (seconds)^b^	0.92 (0.71-1.19)	0.78 (0.66-0.93)	1.06 (0.91-1.23)	0.95 (0.74-1.21)
OARSI^c^ Grade 3 (severe)	1.87 (0.92-3.79)	1.28 (0.79-2.06)	1.60 (1.07-2.39)	2.49 (1.26-4.91)
Grade 1–2 (narrowed)	1.27 (0.73-2.22)	1.08 (0.74-1.56)	1.15 (0.84-1.58)	1.17 (0.73-1.86)
Grade 0 (normal)	1.0	1.0	1.0	1.0

^a^Models stratified by obesity levels. ^b^Odds ratios are per one standard deviation change in KOOS-QOL scale and chair stand test. ^c^X-ray evidence of joint space narrowing.

## Discussion

Persons with BMI levels of at least 25 kg/m^2^ had greater prevalence of severe joint space narrowing, greater pain, and reduced quality of life relative to persons with BMI < 25 kg/m^2^. Despite a greater disease burden among persons with BMI ≥ 25 kg/m^2^, we observed less CAM use and greater conventional medication use relative to those with BMI < 25 kg/m^2^. Our study was consistent with general population studies in that use of individual CAM modalities were less common in those with higher BMI levels, albeit differences were modest [13]. Chiropractic use did not differ substantially by levels of BMI, which was not consistent with studies showing less chiropractic use among obese persons [29]. Considering all individual CAM modalities, CAM use, either alone or in conjunction with conventional medications, was less common among persons with higher BMI levels. We acknowledge that the clinical relevance of some of the observed differences is unclear.

The correlates of CAM use differed by BMI level for some, but not all factors. Women were more likely to report use of CAM for knee OA than men and quality of life was inversely associated with CAM use. Age of at least 65 years was associated with CAM use only among those with BMI ≥ 35 kg/m^2^. Black participants tended to have lower odds of CAM use relative to white participants among those with BMI between 25 and <30 kg/m^2^. This association was not evident among other BMI levels. Our finding that black participants had reduced odds of use of CAM therapies than white participants was consistent with some previous studies [30]. The reasons for racial differences in CAM use are likely multifactorial, including different access to health care and socioeconomic positioning. In our study, black participants had less favorable socioeconomic positioning relative to white participants. Residual confounding from socioeconomic status may partially explain the inverse association. The reasons for inconsistent association between race and CAM use across BMI levels are unclear. Among persons with BMI < 25 kg/m^2^, depression was inversely associated with CAM use. We are unable to evaluate the extent to which these findings are consistent with the literature because, to our knowledge, no BMI level-specific correlates of CAM use have been published.

Given there is no cure for OA, the clinical implications of these findings must be considered. The use of CAM to slow disease progression is not supported by research, yet symptom relief among OA patients has been reported with glucosamine [11] and acupuncture [12]. Obesity is a modifiable risk factor for the development and treatment of knee pain [4]. Indeed, evidence from trials [5-7] suggests that intensive exercise and weight loss interventions result in improved pain and physical function measures. However, the beneficial effects of weight loss may be challenged by joint damage and chronic pain from OA, which cause muscle atrophy, decreased mobility, poor balance, and eventual physical disability [4]. Some researchers have suggested that adoption of weight loss strategies may be hampered by pain, and that intensive treatment of pain resulting from knee OA may improve exercise capacity and quality of life [4].

The reasons why use of the treatment options is less among persons with higher BMI levels are likely multifactorial, and may include differences in patient preference, knowledge, and access to CAM. As the OAI did not collect such information, we were unable to explore the extent to which these factors explained observed differences. Reports show CAM users in general pursue generally healthy lifestyles [31], but use of CAM for weight loss remains relatively low [32]. The latter finding may suggest that persons with higher BMI levels may be reluctant to use CAM in general, rather than reluctant to use CAM specifically for OA symptom relief.

This study has several important limitations to consider. The data shown are cross-sectional. The temporal sequence of symptoms and treatment cannot be determined from this design. Data regarding symptoms and treatment are based on self-report and may have introduced bias. However, the information regarding conventional medications and CAM therapies are based on either a 30-day or 6-month recall. We believe this type of misclassification is likely minimal and non-differential with respect to BMI levels. Thus, if any distortions were introduced, they would have attenuated the observed associations. We are unable to comment on the extent to which patterns of CAM use by BMI levels indicate overuse of CAM by persons with lower BMI level or underuse of CAM by persons with higher BMI levels.

## Conclusion

Our study suggests that despite increased burden of disease, overweight and obese patients with radiographically-confirmed knee OA are using CAM therapies less often than those with lower BMI levels, but use of conventional medications are more common in overweight and obese patients. Overweight and obese adults may be less likely to use effective CAM therapies relative to persons with BMI < 25 mg/k^2^. Further research is needed to improve our understanding of the role of CAM in the treatment of knee OA among overweight and obese persons.

## Abbreviations

BMI: Body mass index; CAM: Complementary and alternative medicine; OA: Osteoarthritis.

## Competing interests

Dr. Eaton has consulted with Pfizer and Dr. Lapane with Janssen. None of the work conducted puts the authors in conflict with the current manuscript.

## Authors’ contributions

KL designed the analysis and wrote the first draft of the manuscript. SY conducted the analysis and interpreted the data. All authors have made substantial contributions to the conception and design and analysis and interpretation of the data. All authors have been involved in the revision of the manuscript for important intellectual content. All authors read and approved the final manuscript.

## Pre-publication history

The pre-publication history for this paper can be accessed here:

http://www.biomedcentral.com/1472-6882/13/241/prepub
